# Actigraphy Measured Physical Activity on Cognitive Functioning in Older Adults

**DOI:** 10.1093/geroni/igab046.3242

**Published:** 2021-12-17

**Authors:** Pilar Thangwaritorn, Amber Watts

**Affiliations:** University of Kansas, Lawrence, Kansas, United States

## Abstract

Physical activity may preserve cognitive functioning in older adults. This study examined associations between objectively measured physical activity and cognitive functioning. We recruited participants (Mage = 75.38 years, SD = 5.99) with (N=26) and without (N=181) cognitive impairment from the University of Kansas Alzheimer’s Disease Center (KU-ADC). We collected cognitive data representing verbal memory, attention, and executive function. Accelerometers (Actigraph GT9X) were used to measure physical activity 24 hours a day for 7 days in a free-living environment. Physical activity was categorized as moderate to vigorous physical activity (MVPA) based on the Freedson (2011) Adult Vector Magnitude cut points. The association between cognitive functioning and total MVPA was evaluated by using multiple regression. We used factor analysis to create three composite scores (verbal memory, attention, executive function) from 11 individual cognitive tests. Compared to verbal memory and attention, results indicate that total MVPA was more strongly associated with executive function (β = 0.001, p = .024). These findings are consistent with the literature suggesting that executive function in older adults may benefit from physical activity. Future research should investigate the physiological mechanisms by which MVPA benefits executive function in contrast to types of activity that might benefit verbal memory and attention.

